# The Effectiveness of Adaptations for Online Remote Public Deliberation Across Three Continents: Mixed Methods Study

**DOI:** 10.2196/59697

**Published:** 2025-09-12

**Authors:** Carly Marten, Emily Bampton, Elin A Björling, Anne-Marie Burn, Emma Carey, Blossom Fernandes, Jasmine Kalha, Simthembile Lindani, Hedwick Masomera, Lakshmi Neelakantan, Swetha Ranganathan, Himani Shah, Refiloe Sibisi, Solveig K Sieberts, Sushmita Sumant, Christine Suver, Yanga Thungana, Jennifer Velloza, Augustina Mensa-Kwao, Pamela Y Collins, Mina Fazel, Tamsin Ford, Melvyn Freeman, Soumitra Pathare, Zukiswa Zingela, Megan Doerr

**Affiliations:** 1 Sage Bionetworks, 2901 Third Ave, Suite 330, Seattle, WA, 98121, United States, 1 2163744633; 2 Department of Psychiatry, University of Oxford, Oxfordshire, United Kingdom; 3 Department of Epidemiology & Biostatistics, University of Washington, Seattle, WA, United States; 4 Department of Psychiatry, University of Cambridge, Cambridgeshire, United Kingdom; 5 Centre for Mental Health Law & Policy, Pune, India; 6 Department of Psychiatry, Walter Sisulu University, Eastern Cape, South Africa; 7 Activate Change Drivers ZA, Johannesburg, South Africa; 8 Department of Epidemiology & Biostatistics, University of California San Francisco, San Francisco, CA, United States; 9 Department of Mental Health, Johns Hopkins Bloomberg School of Public Health, Baltimore, MD, United States; 10 Executive Dean's Office, Faculty of Health Sciences, Nelson Mandela University, Gqeberha, South Africa

**Keywords:** young people, qualitative research, public deliberation, deliberative democracy, data governance, online, remote, MindKind Study, digital divide, mental health data

## Abstract

**Background:**

Public deliberation is a qualitative research method that has successfully been used to solicit laypeople’s perspectives on health ethics topics, but it remains unclear whether this traditionally in-person method can be translated to the online context. The MindKind Study conducted public deliberation sessions to gauge the concerns and aspirations of young people in India, South Africa, and the United Kingdom with regard to a prospective mental health databank. This paper details our adaptations to and evaluation of the public deliberation method in an online context, especially in the presence of a digital divide.

**Objective:**

The purpose of this study was to assess the quality of online public deliberation and share emerging learnings in a remote, disseminated qualitative research context.

**Methods:**

We convened 2-hour structured deliberation sessions over an online video conferencing platform (Zoom). We provided participants with multimedia informational materials describing different ways to manage mental health data. We analyzed the quality of online public deliberation in variable resource settings on the basis of (1) equal participation, (2) respect for the opinions of others, (3) adoption of a societal perspective, and (4) reasoned justification of ideas. To assess the depth of comprehension of the informational materials, we used qualitative data that pertained directly to the materials provided.

**Results:**

The sessions were broadly of high quality. Some sessions were affected by an unstable internet connection and subsequent multimodal participation, complicating our ability to perform a quality assessment. English-speaking participants displayed a deep understanding of complex informational materials. We found that participants were particularly sensitive to linguistic and semiotic choices in the informational materials. A more fundamental barrier to understanding was encountered by participants who used materials translated from English.

**Conclusions:**

Although online public deliberation may have quality outcomes similar to those of in-person public deliberation, researchers who use remote methods should plan for technological and linguistic barriers when working with a multinational population. Our recommendations to researchers include budgetary planning, logistical considerations, and ensuring participants’ psychological safety.

## Introduction

Public deliberation is a community engagement method stemming from (and at times used synonymously with) the political theory of deliberative democracy [1]. Public deliberation, through purposeful provision of information, aims to generate “a discussion that is informed, value-based, and transformative” [2]. Public deliberation engages participants in iterative dialog around complex ethical issues [1]. Public deliberation is used in the biomedical space in contexts such as biobanking [3], genomic research [4], and childhood vaccinations [5], and it differs from focus groups in that intentional information and facilitation are provided to participants to produce dialog, leading to consensus or well-reasoned policy positions [6]. Public deliberation has been traditionally conducted in person, and online public deliberation is an emerging adaptation of this method, which was inspired in earnest by the COVID-19 pandemic [7,8]. Given the novelty of this adaptation, particularly for a high-interaction methodology, such as public deliberation, open questions remain regarding how to engage with participants across the digital divide and how to remotely provide comprehensible information. Furthermore, additional approaches may be needed to assess the quality of deliberation when adapted for a remote audience [7], especially given the concerns of deliberative practitioners that the online environment may lend itself to more uncivil discourse, leading to low-quality engagement [9].

The MindKind Study was a mixed methods international collaboration to investigate the feasibility of a global databank to derive mental health insights [10]. The MindKind Study included a quantitative assessment that recruited participants to collect their mental health data via a mobile app and a qualitative public deliberation assessment that was conducted in concert at sites in India, South Africa, and the United Kingdom. Given the use of public deliberation in biological databanks [1,11] and young people’s rights online [12], we saw this methodology as an appropriate vehicle to educate young adult participants (aged 16‐24 years in the United Kingdom and 18‐24 years in India and South Africa) about data governance and solicit their preferences. In light of the COVID-19 pandemic, we rapidly transformed a method usually held in event spaces over the course of 1 or multiple days [13] into an online, synchronous deliberation coupled with asynchronous dissemination of multimedia informational materials.

In this paper, we discuss the adaptations that the MindKind Consortium made to the public deliberation method in order to inform participants and conduct deliberative sessions online. We also demonstrate our efforts to evaluate the effectiveness of these adaptations, including obtaining evidence of informational material comprehension and assessing the quality of deliberative sessions [14].

## Methods

### Ethical Considerations

The MindKind Study was approved by relevant institutional review boards and ethics boards in the United States (WIRB #20212067), United Kingdom (University of Cambridge - Department of Psychology Research Ethics Committee: Ref. PRE.2021.031; University of Oxford: Ref. R73366/RE00), South Africa (Walter Sisulu University: #029/2021; Department of Higher Education and Training), and India (India Law Society: #ILS/242/2021), as well as by the Health Ministry Screening Committee in India. Potential participants were directed to the enrollment website [15], where they could access the website-based informed consent. The informed consent detailed the privacy and confidentiality procedures for the project, which included anonymous participation and disclosures of the use of the data for research purposes. South African participants were provided with data/airtime, a system to access the internet, to enable their participation.

### Study Design

We recruited young people aged between the minimum age for consent to research as an adult (16 years in the United Kingdom, and 18 years in India and South Africa) and 24 years. We selected these countries for the full study [10] in order to explore the impact of variable high-, medium-, and low-income settings on study results. We held public deliberation sessions in 2 rounds. The first round included participants of a shared nationality, and the second included multinational participants. This design was chosen to build participant confidence in a more familiar setting prior to placing participants of mixed nationalities together.

The topic of deliberation was young people’s preferences for the management and sharing of mental health data (broadly termed “data governance”). Consistent with other online public deliberation studies that have reduced the total deliberation time to avoid “Zoom fatigue” [7], discussion sessions were limited to 2 hours each. In another adaptation from a traditional in-person deliberative model that includes a facilitator, who guides the discussion, and an expert researcher, who serves as a content expert [13], facilitators in this study were trained to answer content-based questions. We combined these 2 roles to ease scheduling constraints and allow for just-in-time sessions, consistent with the participation patterns of young people.

As the provision of informational materials is a key component of public deliberation, the creation of these materials was an intentional and multiphasic process. We iteratively developed informative materials that could be downloaded, rather than live-streamed, by participants in low-bandwidth settings. We adapted a traditional PowerPoint presentation format to the digital environment by interspersing animations and other visual tools to maintain engagement. The basis for these materials was prior work [16] on models of data governance that maximized openness to researchers of a prospective global mental health databank. We solicited feedback from a panel of researchers and technologists, distilling each model of data governance into a description of its function, a use case featuring its application, and a set of advantages and disadvantages of its use.

**Figure 1.**
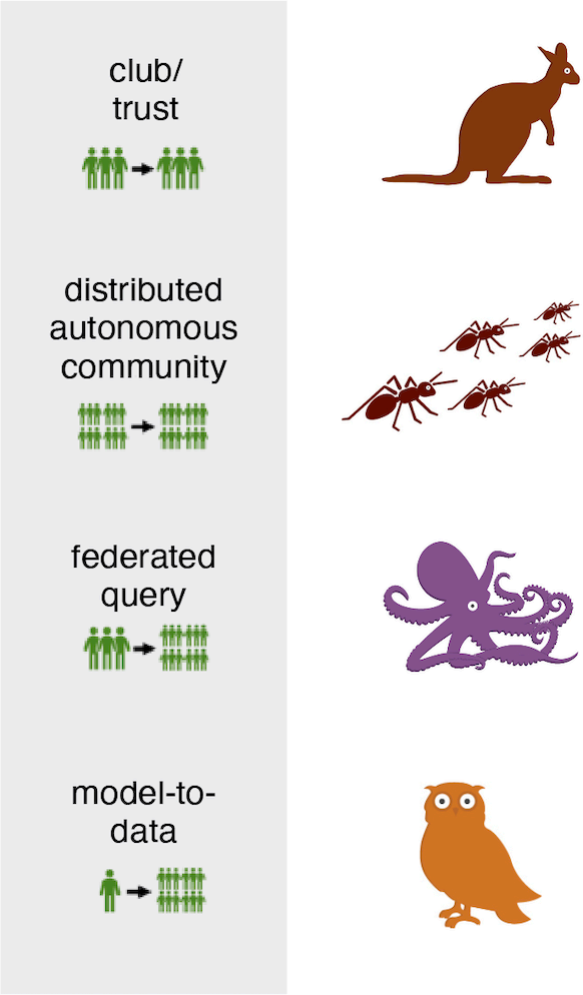
Data governance models described to participants and their animal representations.

We then undertook a process of plain language adaptation. In addition to ensuring that language was at an eighth-grade reading level or lower, we renamed the technical terminology for each model to an animal that exhibited the characteristics of the model. For instance, a distributed autonomous community model was termed the *ant model*, representing how a community is capable of major advances when community members work together. During the plain language adaptation process, we consulted with youth panels to ensure that language was accessible and that animal representations were culturally relevant. Each animal and its model equivalent are provided in Figure 1. For Indian participants, informational materials were translated into Hindi, Marathi, and Tamil.

The resultant informational materials included a 2-module video series with narration by a study team member on each of the India, South Africa, and UK teams. All informational materials are available in an open-access repository [17]. Additionally, inspired by a project at the Open Data Institute [18], we developed an interactive concept map [19] to offer participants a more tactile way to engage with these materials.

An exit survey, hosted on Qualtrics [20], was offered to all participants at the conclusion of the session. The questions were adapted from the study by De Vries et al [14], with the aim of measuring the quality of the remote deliberative sessions. We analyzed four metrics of quality: (1) equal participation, (2) respect for the opinions of others, (3) adoption of a societal perspective, and (4) reasoned justification of ideas. Our exit survey also contained an open response question (“Please use this space to share any additional thoughts.”). We analyzed these data using content analysis [21].

## Results

### Overview

In total, 143 people aged 16‐24 years participated in the public deliberation study (46 from India, 52 from South Africa, and 45 from the United Kingdom), and 61 of these attended multinational sessions. The qualitative results of the full study are detailed elsewhere [10]. Insights on the process of public deliberation in an online, variable resource context are presented below. We developed four types of insights with regard to remote public deliberation: (1) influences of the medium on participation, (2) evidence of informational material comprehension, (3) participant sensitivity to semiotic and linguistic choices, and (4) assessment of quality.

### Influences of the Medium on Participation

Participants used a range of communication modalities within the session, including audio/video participation, writing in the online video conference chat, virtual hand raising, and using the “thumbs up” function and other emoji reactions. At the South African site, participants also added comments over WhatsApp when they experienced a loss of internet connection, and the strength of the participants’ connections often varied throughout the length of the session. Participation was hampered by technical and connectivity barriers at all 3 sites, but most profoundly at the South African site. All 8 in-country sessions in South Africa were affected by participant connectivity, and 2 of these were affected by the loss of facilitator connectivity as well. Participants’ sound quality was frequently compromised by background noise, connection deterioration, or mistakes with the “mute” function. At times, participants who joined the online video conference did not respond to multiple requests by the facilitator for comment, perhaps engaging in other activities instead. As participation was voluntary, participants’ attendance at the session was sufficient to provide a gift card (India and the United Kingdom) or data package (South Africa) incentive, which may have influenced why some participants chose to multitask.

Conversely, the advantage of remote data collection was the ability to safely conduct deliberative sessions in a pandemic context. In our sample, there were participants with clinical vulnerability to COVID-19 and participants who were caretakers for others who may have been excluded from in-person sessions. Facilitators also noted the utility of working from home when sessions were conducted outside regular working hours. The remote approach enabled us to reach participants in geographically distinct locations, both within a given country and in multinational sessions. Participants shared positive reflections on the opportunity to talk to people from other countries. In a multinational session that was live translated for participants of different linguistic groups, a participant shared the following at the end of the session:

*[It] felt very nice, that is we got to do something new and that we are attending the international meeting for the first time. We had a problem with English [.] but still, the opinion of all of us turned out to be similar, and it felt very nice to have a meeting with you. I feel that we are like a family. Thank you.*

[Multinational session 2; translated to English]

Indeed, while the session was logistically challenging to plan and execute, it was well-received by participants and provided unique insights for the research team.

### Evidence of Informational Material Comprehension

We found strong fidelity across participants in relation to the informational materials. Our qualitative results indicated that these materials were, in general, widely consumed, widely understood, and accurately reiterated by participants. Participants made direct references to viewing the informational materials:

*I think, for me, [the option of having a] government [take on the cost of data management] is a “maybe” because if government pays for something, then they have the right to betray us, like in the first module, I saw the government of a certain country betrayed them and shared their information….*

[South Africa session 3; Participant A]

*Funny thing is, she just said it the way I was planning to say because watching those videos. Simply says everything.*

[South Africa session 3; Participant B]

The participants in this exchange recalled an example used in the first video module of TraceTogether, a COVID-19 tracking app created by the Singapore Government that generated controversy when the government shared some location information with the Singapore police force, despite publicly claiming that they would not do so [22]. Participants demonstrated not only a recollection of the details of this example but also an application to their own context, considering whether such an event could occur in South Africa.

Participants exhibited a command of the complex details of data governance models presented to them:

*I chose the octopus model as my favorite one. I mainly liked the controls over the sharing of the data in the sense that, with the example one that was in the video, showing that people were…able to access a base level of data just online, so anyone have that access, but for specific research access, it was more involved with what they wanted to do with it…I guess the main issue with that model, though, is the fact that because it is so decentralized and it might be hard to know what you’re going to need to provide when you’re trying to access that data, because, say, if it’s all from different groups, they might all have different requirements.*

[UK session 1]

This quote referencing a federated query data governance model and the example of Beacon Network [23], a search platform for genetic variants, demonstrates a sophisticated recollection of informational material details. This participant not only accurately described the way in which Beacon Network functions but also went on to appraise this system (“I guess the main issue”), indicating how the participant is applying their learnings.

Participants, at times, may have made mistakes in their recall of informational materials but retained understanding of their core messages:

*[W]hen we’re talking about research, I am constantly thinking about the example that owl model had.... [W]hen we give access to everyone, somewhere, what the results of these kinds of researches will be, will also be accessible by everyone, and then how people, you know, take this information and what they do with it, and how they present it later, will then be to their discretion. And when the general public sees that information, they’ll believe it, irrespective of whether that person has the skills to even, you know, work on that data in the first place or not.*

[India session 4]

The participant directly referenced the *owl* model, which was the animal term for a model-to-data governance scheme, wherein researchers submit computational models that are run on a private dataset. The example used in relation to this model was the National COVID Cohort Collaborative’s research on the predictors of COVID-19 infection [24]. Because creating computational models requires sophisticated programming skills, it is not quite accurate that “everyone” has the ability to engage with the electronic health record data in this use case. It may be that the participant is referring to a more democratizing data governance model, such as a distributed autonomous community model (what we called the *ant* model), which does enable collective data ownership and use by citizen scientists [25]. Regardless, this quote demonstrates how even participant recollections with putative errors are still usable to qualitative researchers. This participant articulated a concern that if unqualified people in the “general public” have access to mental health data, they could use this information at “their discretion,” potentially nefariously. Ultimately, speaking mistakes were of little consequence because the facts upon which participants made value judgments were well understood.

### Participant Sensitivity to Semiotic and Linguistic Choices

**Figure 2.**
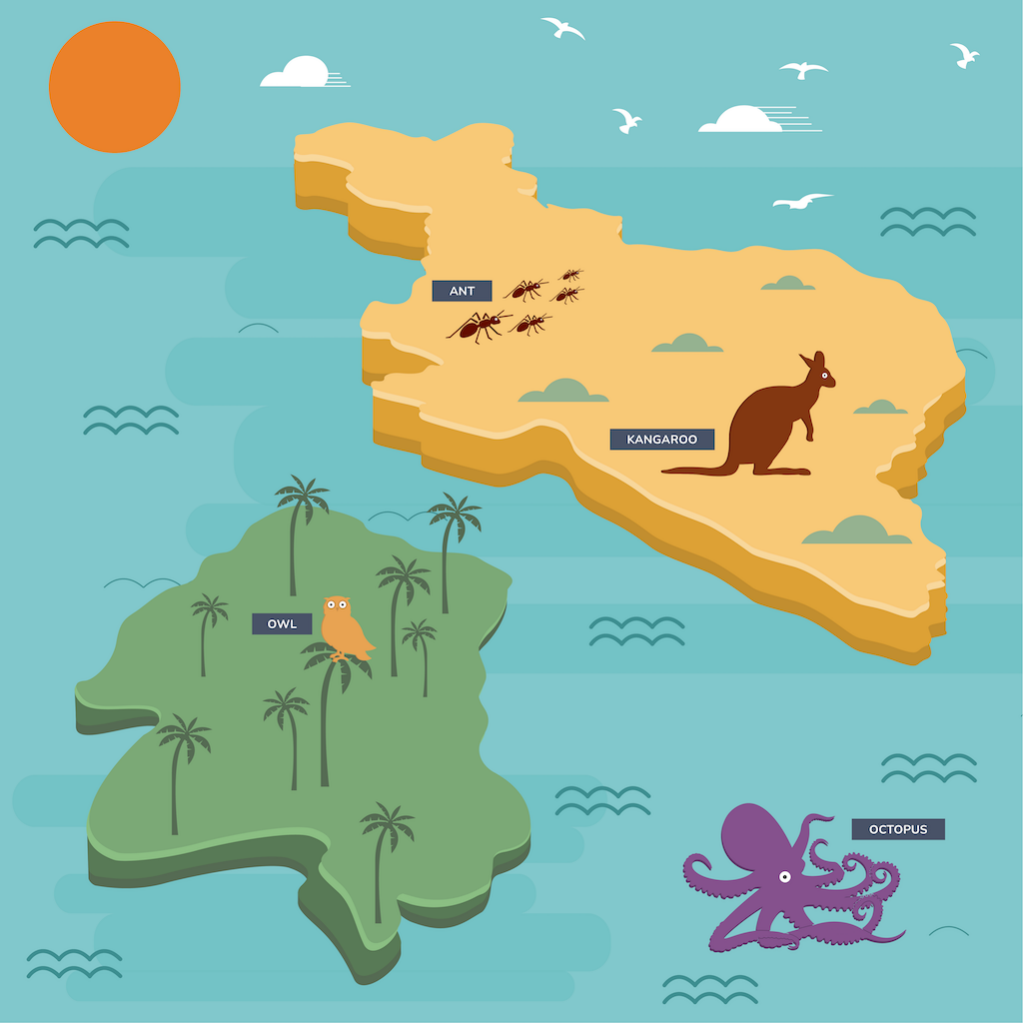
Original draft of the interactive concept map image.

Despite the strong overall understanding of participants, we want to share some specific findings that demonstrate participants’ awareness and sensitivity to choices made in informational material development. Prior to data collection, we tested an image that was to form the basis of our interactive concept map with youth panelists (Figure 2).

Although the animal images (ant, kangaroo, owl, and octopus) are specific touchpoints on a webpage that users can select to learn about a given governance model further, the background scenery (water and landmasses) was designed to be insignificant. However, youth panelists thought that the positioning of the animal touchpoints communicated the similarity and dissimilarity of animal models to one another. Panelists gathered that the *ant* and *kangaroo models* were uniquely similar because they shared a landmass, although this was merely a design choice. This finding indicated to the research team both the utility of testing informational materials prior to deploying them and the possibility for participants to glean information from unintentional semiotic signifiers in the materials.

During data collection, we also observed participants’ sensitivity to particular linguistic choices. The cornerstone of our informative materials was a set of data governance choices presented in a 7-question data governance typology [10], which was the product of extensive iteration and plain language adaptation. In response to the question *Who controls the data?*, participants were offered the option of *community hires a manager*, which refers to a community of participants, users, or researchers using a data steward to manage a database. While a data steward can be a single individual, it is more often a group of individuals working for an organization. However, this language (*community hires manager*) was frequently understood by participants to indicate a single individual managing a database. As such, participants indicated a fear of undue concentration of power in such a manager:

*I don’t think you can ever trust one person, especially with global data. It’ll put too much pressure on them...also, it’s just one person,...they don’t have the same views as everyone else who also wants to be able to control the data or know what’s happening with the data.*

[Multinational session 1]

*I really don’t like the idea of a manager because there are bad eggs everywhere and you don’t want to give* one person *that amount of power*.

[UK session 4]

Accordingly, even in light of extensive testing of this typology, there were still unknown signifiers in the language we used that could influence participant preferences.

### Assessment of Quality

We obtained 159 exit survey responses (40 participants who marked their country as India, 38 who marked South Africa, 52 who marked the United Kingdom, and 29 who marked multinational). Each survey response does not represent a unique individual (there were 143 in this study) because, following multinational sessions, wherein all participants were sourced from earlier in-country sessions, participants were directed to the same survey. Despite our efforts to distinguish the in-country responses from the multinational responses by asking participants to select *multinational* as their country, many participants in multinational sessions still selected their home country, making it difficult to disentangle in-country session responses from multinational session responses.

### Equal Participation

While De Vries et al [14] measured the volume of text contributed by each participant, the multimodal ways in which participants contributed to our study make this a challenging metric to replicate. Coupled with participants who joined late, left early, or experienced technical difficulties, we did not feel that the volume of text was a meaningful measurement in our case. Instead, we are reporting our facilitator training strategy for ensuring equal participation. Facilitators were instructed to solicit the opinions of quieter participants and to seek approximately equal participation of the 3 countries in multinational sessions. Facilitators directly solicited participants with statements like “I would be interested to hear [name]’s thoughts on this” or “Does anyone from South Africa have an opinion to share?” Another effective strategy was assigning a number to each participant and making a request like “Let’s hear from the even numbers” when conversation became stilted. Despite our best efforts, it was challenging to obtain true equal participation in this context.

### Respect for the Opinions of Others

Our metric for the item is adapted directly from the study by De Vries et al [14]. It asked “Do you feel your opinions were respected by your group?” (response on a scale ranging from 1 [not at all] to 10 [very much]). The average score in the study by De Vries et al [14] was 9.4 (SD 1.0). Our score was similar at 9.6 (SD 1.0; median 10, range 1-10; n=150) (Multimedia Appendix 1). We also replicated the next question from the study by De Vries et al [14] on the same scale: “Do you feel that the process that led to your group’s responses was fair?” Again, the results were similar. De Vries et al [14] found a mean score of 9.7 (SD 0.7) [14], and we found a mean score of 9.5 (SD 1.0; median 10, range 5-10; n=143) (Multimedia Appendix 2).

### Adoption of a Societal Perspective

We adapted the inquiries in the study by De Vries et al [14] for the metric. De Vries et al [14] inquired at different time points whether participants would allow a surrogate to decide to enroll them in a gene transfer study (54% affirmative immediately following the study) and whether participants would use surrogate consent to enroll a loved one in a gene transfer study (41% affirmative immediately following the study). We asked two adapted questions at a single time point immediately following the study: (1) If a global mental health databank was created according to the specifications your group chose today, would you contribute data about yourself? (2) If a global mental health databank was created according to the specifications your group chose today, would you recommend that your community contribute data about themselves? The “yes” response rates for questions 1 and 2 were 91% and 93%, respectively.

Notably, our “yes” response rates are considerably higher than those in the study by De Vries et al [14], which might be attributable to the relative clinical invasiveness of a gene transfer study as compared to an informational databank study.

### Reasoned Justification of Ideas

Participants shared richly reasoned arguments for why various data governance schemas were or were not acceptable to them. Sessions were not without “Because I said so” justifications, as defined by De Vries et al [14], but facilitators were trained to ask follow-up questions, as exhibited by this exchange:

Participant: *[in response to the question Who controls the data?] Okay. So I would say no one [controlling the data] is acceptable.*

Facilitator: *Could you elaborate on why?*

Participant: *I say no one is acceptable because if you meet the requirements in whatever process you have to undergo, then it means you simply qualified [.] and the information should only be given or not given. It should be accessible to people with the necessary qualifications to access the information.*

[South Africa session 8]

There are methodological reasons why a participant may not initially share a fully reasoned response, such as the limited time for discussion and the awareness of consensus-building as a goal. As demonstrated by the open-text responses below, wherein participants reflected on the value of hearing from others, participants warmly received the discussion aspect of the session, suggesting the richness of the interpersonal communication displayed.

In response to the open-text question, respondents shared broadly complimentary comments on the research process. Some shared recommendations to improve the participant experience of data collection:

*Make a document that the group can communally edit (ie google slides*)

[UK session participant]

On the other hand, some reflected on the utility of a mental health databank in general:

*Data about mental health and mental health related studies should be accessible to students and s researchers [sic] just for the purpose of understanding the community better, providing them better help and doing better by the people.*

[Indian session participant]

Many commented on the value of the discussion experience itself:

*I felt really heard and that everyone had the opportunity to speak and share their thoughts. I feel like it is so important for people to be involved in these conversations. The call was really interesting too and the hosts ensured the atmosphere was welcoming*.

[UK session participant]

*[A]s an individual coming from a country that is vastly different from those within the meeting, there were many commonalities that we were able to decide on during the session. [S]ome topics did require more of a discussion and debate, while others were collectively decided*.

[Multinational session participant]

As demonstrated in the quotes above, among participants who shared an open-text response, their comments reflected engagement and willingness to continue the conversation.

## Discussion

### Principal Findings

The qualitative arm of the MindKind Study offers an example of remote, online public deliberation with participants in varied geographies. Other deliberative studies have provided video-based informational materials to participants [26], including young people [27], with similar success. Our research team may have benefited from an approach we adopted during the development of informational materials by trying to optimize project tools for the online environment (eg, by using animations, an interactive concept map, and emoji reactions on video conferencing platforms) rather than trying to mirror the in-person experience more closely. However, this undertaking was not without limitations.

Participation was hampered by barriers due to time zones, technology access, and language challenges. Multinational sessions, for instance, were only conducted within a limited time window to allow participation from 3 distinct time zones. As such, participants who were unable to connect during this period due to school or work commitments could not join the study. Indeed, as articulated by Bulling et al [28] in an overview of deliberation models involving young people: “Many youth schedules are tighter and more inflexible than those of the decision-makers who hope to involve them.”

We chose to limit the deliberative session time to 2 hours, which is consistent with other online public deliberation studies, but we did not ask participants to return for multiple weeks of ongoing meetings in the way that other remote deliberation studies requested [7]. At most, participants engaged in 4 hours of deliberative sessions in total if they attended both an in-country and a multinational session. While online public deliberation studies in high-resource countries have been able to obtain a high retention rate across several deliberative sessions (such as 91% across 5 sessions in Canada [29]), we struggled to retain many South African and some Indian participants across just a 2-hour timeframe due to variable connectivity.

Given that a traditional in-person deliberative study is performed over a multiday period [13], there are substantive questions of whether online deliberation, especially in low-resource contexts, truly approximates in-person data collection. The online environment may not lend itself to the collective, focused experience achieved in an in-person setting [8]. Furthermore, young people lacking a device connected to the internet were unable to join the study, and participants with a weak network connection may have experienced less meaningful interactions than others. While we did not provide device loans to participants as other deliberative researchers have [8], we provided data packages to participants in South Africa to counter the high costs of data for connectivity, which we found to be highly influential. However, when the network infrastructure itself poses barriers to connectivity, such as ongoing rolling blackouts in South Africa, there may be little that researchers can do to account for this effect.

Public deliberation practitioners have also expressed concern about the balance of power in online deliberation, potentially leading to degraded quality of conversations and even perversion of results [9,30]. We were particularly concerned about this effect across postcolonial contexts, which is why we implemented in-country deliberation prior to multinational deliberation. While the results of our assessment of quality [14] are promising, we acknowledge that this is an imperfect tool for our context, especially in light of the digital divide, which may have a heightening effect on social inequality [30]. We encouraged facilitators to practice awareness of social dynamics on deliberative quality, but an assessment tool that is better suited to an online, variable resource setting would be beneficial.

Some concepts in the informative materials were particularly challenging to explain, especially without an in-depth dialog with participants, as is customary in an in-person research setting where participants can direct questions to expert presenters [13]. Similar to the findings of Lemke et al [3] with regard to educating participants on the concept of a “biobank,” this study also exposed participants to terminology and concepts that were novel to them. The explanation of the concept of a synthetic dataset [31], which we termed a *recreated dataset*, was persistently challenging for both participants and facilitators. This had been evident since the testing phase of the materials, and we attempted several analogies and representations with youth panelists, which were not well-received. Participants often expressed concerns that a recreated dataset would not accurately capture the underlying data, which is a legitimate concern in the research literature [32].

A more fundamental shortcoming of these materials was related to their accessibility to non-English speakers who spoke 1 of 2 regional languages in India or one or more of a mix of Indigenous South African languages. While site teams in India, South Africa, and the United Kingdom perceived high levels of understanding among their English-speaking participants (mixed first- and second-language English speakers), the materials were not as successful among non-English–speaking participants. Facilitators in India noticed substantive differences in the nature of clarifying questions between English-speaking and non-English–speaking participants, with the former asking questions about sophisticated research processes and the latter asking more fundamental questions about concepts around data and research. Facilitators needed to make rather unrelated analogies that were relevant to participants’ everyday lives to bridge the understanding gap.

There are a few potential reasons for this discrepancy. The original copy of the informational materials was written in English, and the materials were based on research concepts largely published and discussed in English. As such, multilingual Indian researchers found these materials to be challenging to translate into regional languages because either equivalent terms did not exist or such terms were not in everyday use to be comprehensible to young people. Moreover, the materials were translated into a more formal register of a given regional language, which the participants found difficult to understand, considering the novelty of the concepts. Additionally, the non-English–speaking participants may have had lower levels of exposure to technology and research in general. Although we tested materials for plain language readability, the concepts presented were still very sophisticated and perhaps better understood by participants with some exposure to research studies, research data, and related technologies. In future studies, it may be preferable to first develop materials in the target language and subsequently translate them to English [33]. Finding language representations, analogies, stories, and semiotic representations that bridge the understanding gap without compromising the integrity of the message is an ongoing challenge for public deliberation researchers seeking to communicate about complex concepts.

### Recommendations

In the context of multinational online remote public deliberation using multimedia informational materials, we present a set of recommendations based on our experience. First, researchers may need to make structural adjustments to their project timelines and budgets to account for remote data collection. Despite the relatively lesser time commitment of a video conference compared with an in-person event, the recruitment of participants and development of informational materials for remote public deliberation are arguably more labor-intensive. Furthermore, researchers should include data reimbursement or data package provisions in their budgets, especially for participants in regions with low internet penetration levels. At the South African site, we found that upfront data package provision was a necessary precondition for most participants to join the study. Correspondingly, researchers should ensure that their teams have sufficient provisions in place to account for a team member losing internet connection during a session.

Researchers working with multinational participants should also take into account participants’ comfort and psychological safety in these settings. In our multinational deliberative sessions, we arranged for 1 research team member from each site to be present, and we developed a language use guide of terminology that could help participants sensitively communicate with peers having different nationalities, language backgrounds, and mental health experiences.

Finally, we were unable to use an online learning management system due to time and capacity constraints. Such platforms may enable researchers to organize materials at a single location, confirm participants’ viewing of materials, and break videos into smaller segments. We encourage researchers to consider and budget for such platforms. Additionally, we recommend that researchers co-develop these materials with representatives from the participant population and make the language as accessible as possible.

### Conclusion

Online remote public deliberation is a useful adaptation of a traditionally in-person research approach, which can enable safe and meaningful multinational participation. However, researchers who use remote methods must attend to technological and linguistic barriers, especially when translating informational materials from their original language.

## Supplementary material

10.2196/59697 Multimedia Appendix 1 Score distribution for the question “Do you feel your opinions were respected by your group?” (response on a scale from 1 [not at all] to 10 [very much]).

10.2196/59697 Multimedia Appendix 2 Score distribution for the question “Do you feel that the process that led to your group’s responses was fair?” (response on a scale from 1 [not at all] to 10 [very much]).
