# Supplementary figures and images for: The Effectiveness of Adaptations for Online Remote Public Deliberation Across Three Continents: Mixed Methods Study
Source: J Particip Med. 2025 Sep 12;17:e59697. doi: 10.2196/59697 (PMC12431157; doi:10.2196/59697)

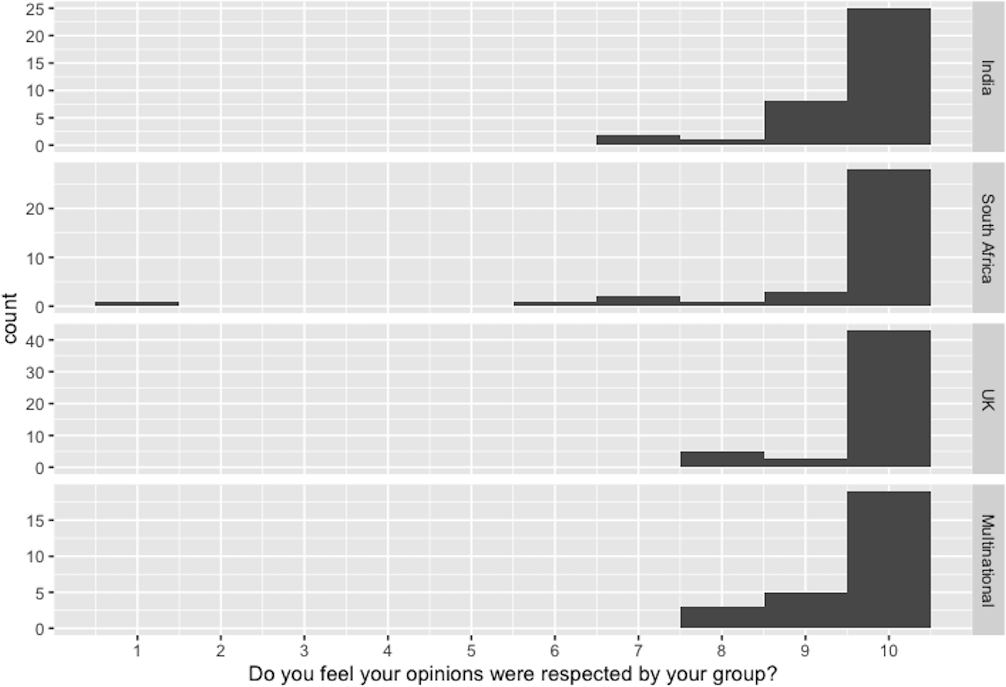

Supplement: Multimedia Appendix 1 [file jopm-v17-e59697-s001.png]

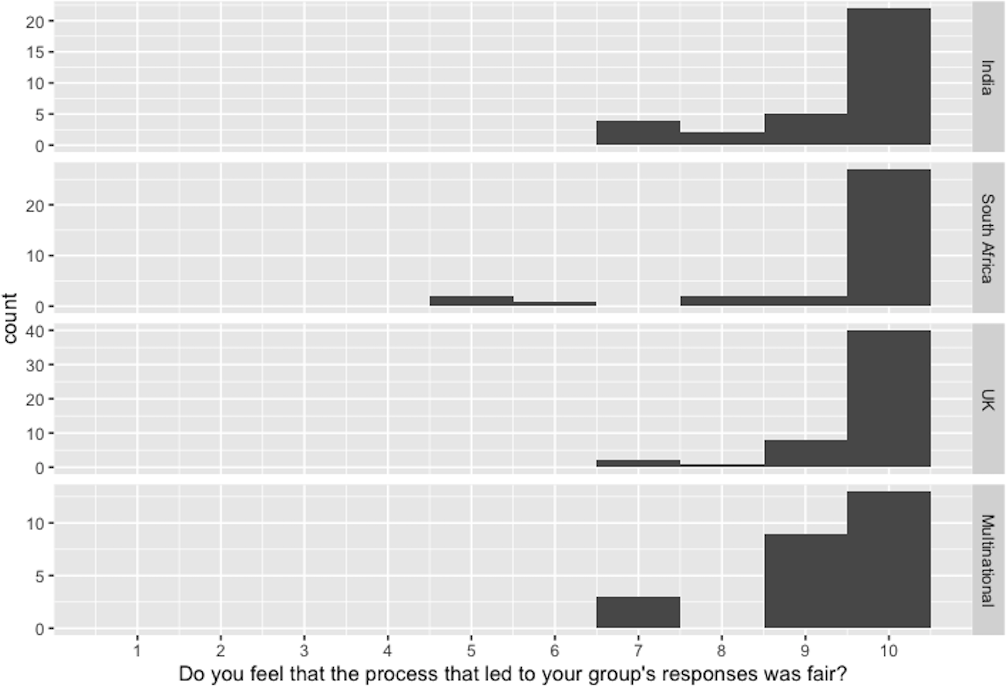

Supplement: Multimedia Appendix 2 [file jopm-v17-e59697-s002.png]
